# Role of nurse informaticists in the implementation of Electronic Health Records (EHRs) at resource-limited settings

**DOI:** 10.12669/pjms.40.9.9686

**Published:** 2024-10

**Authors:** Kiran Bano Asif, Haroon Khan

**Affiliations:** 1 Kiran Bano Asif, Nurse Informaticist, CMIO Office, The Aga Khan University Hospital, Karachi, Pakistan; 2 Dr. Haroon Khan, Physician Director, CMIO Office, The Aga Khan University Hospital, Karachi, Pakistan

**Keywords:** Nurse Informaticists, Electronic Health Records (EHR), Healthcare Technology, Low middle-income countries (LMICs), EHR Implementation, Health Information Technology (HIT), Healthcare Informatics

## Abstract

Electronic health records (EHRs) play a critical role in the management of patient information and timely decision making in health facilities. In resource-limited settings, especially low- and middle-income countries (LMICs), nurse informaticists play a pivotal role in the implementation of EHRs. This article underscores their multifaceted responsibilities, emphasising critical contributions in vendor selection, system evaluation, workflow analysis, content development, end-user device assessment, training, and post-implementation stability support. By providing nurse informaticists in lower middle-income countries with a clear understanding of their responsibilities and tailored strategies, this article aims to enhance EHR implementation success in these unique contexts.

## INTRODUCTION

In 1992, the American Nurses Association (ANA) identified nursing informatics as a specialty that integrates nursing science with multiple information and analytical sciences to identify, define, manage, and communicate data, information, knowledge, and wisdom in nursing practice.^1^ Nurse informatics professionals play a vital role in all aspects of the health information technology (HIT) lifecycle. According to a 2020 workforce survey from Health information and management systems society (HIMSS), the most common job duty reported by informatics nurses is system implementation or electronic health records (EHR) implementation, followed by system optimisation, utilisation, system development, project management, quality initiative planning, reporting, and education of others on informatics.^2^ EHR is a data repository and clinical information accumulated in electronic health records (EHR) is used in many secondary settings, such as quality, legal, and regulatory venues, and the data is analysed to produce new nursing knowledge as well. 3

In resource-limited settings, especially in low- and middle-income countries (LMICs), implementing electronic health records (EHRs) goes beyond technology adoption. In LMICs, where resources are scarce, effective use of EHRs holds transformative power, enhancing patient care and serving as essential tools for quality improvement and regulatory compliance.^4^ Here, the crucial role of nurse informaticists becomes evident. This article explores the multifaceted responsibilities of nurse informaticists, highlighting their important role in successfully implementing EHRs, with a focus on the unique challenges and adaptations required in LMICs.

### Vendor Selection and System Evaluation:

An important element of a nurse informaticist’s job is to ensure that new systems integrate seamlessly with existing hospital systems and the nursing workflow.^5^ The success of health information technology (HIT) implementations is often tied to the impact the technical system will have on the work of the end users using it. A well-designed system that supports the processes within the culture of an organisation is the key to successful implementation.^6^

The implementation of an EHR may be one of the largest projects a healthcare organization undertakes. After thorough assessment and planning, when the decision is made, the next step is to explore and select a vendor that meets the needs of the practice. The role of the nurse informaticist at this stage is crucial, it involves researching and comparing different EHR vendors and their capabilities to implement the system. with those of other team members. This phase requires astute guidance from nurse informaticists, especially in LMICs, where resource constraints demand judicious decision-making. It involves a comprehensive evaluation of various EHR vendors and their capabilities in collaboration with the team. This could include visiting vendor websites, attending demos and webinars, researching previous client successes and challenges, and getting references from other organisations that have implemented similar systems.^1^ In LMICs, this process demands an even more robust strategic approach, considering factors such as cost-effectiveness and adaptability to limited technological infrastructure. On the other hand, they should remember that the goal of EHR implementation is to improve documentation accuracy, eliminate unnecessary work, and enable the analysis of clinical data.

### Self-Training:

Nurse informaticists need to be familiar with the EHR they are implementing to effectively troubleshoot issues that arise during and after implementation. They should also need to be able to train other nurses and staff on how to use the system effectively, which can improve adoption rates and ensure that the system is being used to its fullest potential. Self-training is an essential part of the preparation for EHR implementation. Research shows that nurse informaticists who engaged in self-training were better able to provide guidance and support to end-users during and after implementation, leading to higher rates of system adoption and better patient outcomes.^6^ Self-training can help nurse informaticists gain a better understanding of how the EHR can be used to improve patient care and outcomes.^7^ In resource-constrained settings, leveraging online resources and tutorials, often available at no cost, allows us to access valuable learning materials. Additionally, vendor-provided training materials, typically offered as part of the EHR package, represent an economical way to gain in-depth knowledge of specific systems. Accessing demo or sandbox environments, if available, enables hands-on practice in a risk-free setting. Furthermore, engaging with online communities and forums centered around EHR implementation offers a platform for knowledge exchange at no additional expense. These strategies empower nurse informaticists to develop their competencies in the smooth implementation of EHRs without incurring substantial financial burdens.

### Coordination with End-Users:

Before a product is developed for healthcare professionals, the software developers need to understand the needs of the users. Clinical nurse leaders and managers can contribute significantly to explaining and coherently stating the requirements so that the product that comes out is not redundant. The involvement of these clinical nurses in the initial phases of software development would ensure a better product that is different and more relevant clinically. 8

Nurse Informaticist coordinates with hospital leadership to form a team of subject matter experts. These subject matter experts are nurses from individual entities, for example, medicine, oncology, and surgery etc. These experts will serve as focal person, content experts, and decision-makers for an individual department. Nurse informaticists ensure the participation of the nurses in the software development process as they understand the clinical flow and patient care processes better. This becomes more relevant as EHRs are now looked upon as not only a source of convenience but a mode to enhance patient care processes and quality improvement.^5^ The involvement of nurses could also address common problems that may arise due to ill-designed software, as well as ensure better allocation of healthcare resources. The need for nurses to be a part of the software development process has long been recognized; therefore, nurses should be involved in the selection, development, and implementation of any system.^9^

### Workflows Review:

The role of nurse informaticists is pivotal in the development and management of workflows for electronic health record (EHR). Their expertise in clinical workflows, healthcare regulations, and technology enables them to bridge the gap between clinical practice and technology, ensuring that the EHR supports safe and effective patient care.^10^

Nurse informaticists participate in workflow analysis to identify steps and processes involved in patient care. This information is then used to develop EHR workflows that support efficient and effective patient care.^11^ They also work with clinical teams to identify areas for process improvement and develop strategies to optimise workflows within the EHR. They also work with EHR vendors and IT (information technology) staff to configure the EHR to support clinical workflows and business processes, which may include customizing documentation templates or developing order sets.^11^

Overall, the function of a nurse informaticist in workflow mapping for EHR is to ensure that the EHR supports safe and effective patient care. This requires a deep understanding of clinical workflows, healthcare regulations, and technology, as well as strong communication and collaboration skills to work effectively with clinical teams and IT staff.

### Content Development:

A robust, complete information system can improve the efficiency and consistency of patient care and reduce errors in documentation and communication. Nurse informaticists have a critical role to play in identifying areas for improvement in quality patient care that can be addressed with information technology (IT).^12^ For example, they can develop standard operating procedures to prevent medication errors and antibiotic resistance with the help of quality improvement nurses who can track these important areas.

Nurse informaticists are responsible for collecting an inventory of the documents currently used by the organization, including tools, checklists, pathways, and order sets. They then work with an IT team and vendor to develop these documents and tools for the electronic health record (EHR), ensuring that they reflect current evidence-based practices and meet the satisfaction of subject matter experts. The development of this content is critical to revolutionizing nursing practice, eliminating tedious and repetitive documentation tasks, and enabling nurses to spend more time at the patient’s bedside.

### Collaboration with IT (Information Technology) team and Vendors:

Nurse informaticists play an essential role in software development for healthcare systems. While software developers may understand the functional requirements of the programme, they may not be familiar with medical terminology or patient care data relevant to the acuity system, which could result in a false clinical picture if not presented accurately.^5^ To ensure accurate communication of patient care information among healthcare providers, it is essential to define a standardized nursing language for patient care systems. Nurse informaticists work closely with the IT team to define and implement standard nursing terminology, outlining the scope of nursing care and reflecting it in healthcare software. 5 Additionally, they collaborate with IT teams to ensure that electronic health records (EHRs) are designed in a way that promotes patient safety and quality care. They work with IT teams to identify and mitigate risks associated with the use of EHR and other health information technologies.^10^

Nurse informaticists can work with vendors to develop customised solutions that meet the specific needs of their organisation. This includes developing software that is tailored to the workflow of the clinical staff and provides real-time information to support decision-making and patient care delivery.^1^

### End User Device Assessment:

End-user devices such as computers, tablets, workstations on wheels (WOW), smartphones, wall mount monitors, dual monitors, etc., These are essential tools for nurses to access and document patient information. Nurse informaticists play a critical role in evaluating end-user devices based on usability, mobility, compatibility, and security. They also work closely with vendors and IT departments to ensure that the devices meet the needs of the end-user and comply with organisational policies and regulations. Engaging nursing and physician teams in the assessment and selection of end-user devices holds particular significance in LMIC. In these settings, resources are often limited, making it essential to make informed and cost-effective decisions. By involving frontline healthcare providers in the process, healthcare facilities in LMIC can ensure that the chosen devices align with their specific needs and workflows while also avoiding unnecessary financial burdens on the institution.

Nurse informaticists also participate in the testing and validation of end-user devices to ensure that they are compatible with the EHR and meet the needs of healthcare providers. This may include testing devices for performance, security, and usability. Based on the results of device testing and validation, they provide recommendations for the selection and configuration of end-user devices. This may include recommendations for hardware specifications, and software specifications to ensure that healthcare providers have the tools and technology needed to effectively and efficiently access and interact with the EHR.

### Testing:

Testing is a critical phase, as a system’s design can significantly impact its acceptability among users. Nurse informaticists, along with end-users, take on the role of test analysts or testers to verify the credibility of software.^9^ They serve as testers, test analysts, and subject matter experts in different types of testing, such as iteration, mock testing, and retesting of content available before the EHR goes live. The involvement of nurse informaticists in these testing phases can help identify any issues related to system functionality, usability, and safety.^10^

As testers, nurse informaticists can evaluate the system’s functionality and user interface to ensure that it meets the clinical requirements and workflows. Nurse informaticists can also serve as test analysts, reviewing test cases and plans, and conducting quality assurance to ensure that the EHR is effective and efficient.

### Staff Training:

The implementation of an electronic health records system requires proper training for end-users, including current and new employees. The training must go beyond teaching the basic functionalities of the system and should address the specific day-to-day work of each user. Training of end users must address the training of end users on future state workflows so that they are aware of new workflows at the time of implementation. In addition, ongoing access to a demo system that contains the full functionality of the EHR should be available to users for continuous practice. Training should not be a one-time or infrequent event but rather a continuing, ongoing part of the education routine for the healthcare organization to keep up with the constant changes in health information technology.^13^ Appropriate space and alternative methods of instruction should be developed to facilitate ongoing training. The use of training development models can provide consistency and successful outcomes while allowing staff to focus on delivering safe patient care.^13^

Nurse informaticists are responsible for designing and developing training programmes that cater to the specific needs of nurses and other healthcare providers, ensuring that they are comfortable with using the new technology and can integrate it effectively into their workflow.^14^ They work with subject matter experts and end-users to develop training materials that are easy to understand and use. They also provide ongoing support to end-users to help them address any issues that arise during the implementation of new technology.^15^

In LMIC, addressing training for the implementation of electronic health record (EHR) requires a context-specific approach. Given potential resource limitations, it is crucial to prioritise cost-effective and sustainable training strategies. Nurse informaticists in LMIC can take a proactive role in creating a dedicated training team within the healthcare facility, collaborating with existing departments such as nursing education services or other education departments.^16^ This team can facilitate training sessions tailored to the specific needs of nurses and other healthcare providers, reducing the reliance on costly vendor-led training programs. Moreover, investing in ‘train-the-trainer’ initiatives and identifying superusers within departments can be a highly effective approach. These superusers can serve as internal experts, disseminating knowledge and providing ongoing support to their colleagues. By leveraging local resources and expertise, nurse informaticists play an important role in equipping healthcare providers with the skills needed to effectively utilise EHRs.

### EHR Go-Live Support:

During EHR go-live, nurse informaticists play a central role in providing support to clinical staff, troubleshooting issues, and ensuring a smooth transition to the new system. They act as a liaison between clinical staff and the IT team, communicating feedback and suggestions to improve the system’s usability and functionality.^13^

Several preparatory steps should be taken to successfully run a new implementation in a hospital or clinic. These involve such stages as healthcare providers’ training and the installation of new hardware. The preparatory process ought to follow a script, which is called the Systems Development Life Cycle (SDLC). It involves stages of planning, analysis, design, maintenance, and implementation.^9^,^17^ A team of professionals should be working together, and various computer systems should be interacting and functioning efficiently with each other. Nurse informaticists, whose key qualities are abilities to communicate, organize, plan, and provide support during the process. 5 They are responsible for conducting pre- and post-go-live assessments, identifying issues and areas for improvement, and providing end-user training and support. Nurse informaticists also play a significant part in ensuring patient safety during the transition to the new system. Additionally, the presence of a nurse informaticist during go-live support is important to address the concerns of clinical staff, guide them on using the new system, and facilitate communication between clinical staff and the IT team.

### Post Go-live Stability Support:

Nurse informaticists in the post-implementation phase participate in offering continuous support and sufficient training. This comprises technology understanding and its impacts to ensure the existence of the new system and its compliance and acceptance. Besides, it also aids in augmenting adherence to and satisfaction with the best practices. Consequently, any probable negative impacts because of technical failure can be mitigated. The involvement of the nurse in the post-implementation stage is important in pinpointing the flaws, subsequently reinforcing the essence of continuous database maintenance and upgrade.^18^

Following the implementation of the system, the nurse informaticist evaluates the results and helps the staff maintain the system effectively. A nurse informaticist examines the practical application of the system and shares relevant information with the developers regarding any necessary modifications. The team collaborates to anticipate any potential issues and devise strategies to prevent them.^5^

## CONCLUSION

Nurse informaticist is involved in implementation of EHR in various stages, including planning, design, and post-implementation. During the planning phase, they work with other stakeholders to identify the organization’s needs and select the appropriate EHR. During the design phase, nurse informaticists focus on optimizing workflows to maximize efficiency and resource utilization, especially in LMIC, considering the diverse patient populations and prevalent healthcare delivery models. During implementation, they participate in go-live support, staff training, and system testing. In the post-implementation phase, they provide ongoing support, evaluate the system’s outcomes, and identify areas that require improvement. Notably, in LMIC, the emphasis often lies on finding sustainable and cost-effective solutions that can be maintained over the long term. Their involvement ensures the successful implementation and adoption of EHRs, leading to improved patient outcomes and more efficient healthcare delivery.
